# Supplementary material for: Chinese Students’ Health Literacy Level and Its Associated Factors: A Meta-Analysis
Source: Int J Environ Res Public Health. 2020 Dec 29;18(1):204. doi: 10.3390/ijerph18010204 (PMC7796290; doi:10.3390/ijerph18010204)
Supplement: Supplementary file 1 [file ijerph-18-00204-s001.pdf]

Appendix

Search strategy

Database 1: PubMed

Search	Query	Items found
**#1**	"Health Literacy"[Mesh]	6024
**#2**	Literacy, Health[Title/Abstract]	177
**#3**	("Health Literacy"[Mesh]) OR (Literacy, Health[Title/Abstract])	6121
**#4**	China[Title/Abstract]	188,705
**#5**	chinese[Title/Abstract]	215,063
**#6**	(China[Title/Abstract]) OR (chinese[Title/Abstract])	357,345
**#7**	((((Student[Title/Abstract]) OR (School Enrollment[Title/Abstract])) OR (Enrollment, School[Title/Abstract])) OR (Enrollments, School[Title/Abstract])) OR (School Enrollments[Title/Abstract])	119,092
**#8**	((((((((pupil[Title/Abstract]) OR (elementary student[Title/Abstract])) OR (middle school student[Title/Abstract])) OR (junior[Title/Abstract])) OR (senior[Title/Abstract])) OR (high school student[Title/Abstract])) OR (undergraduate[Title/Abstract])) OR (academician[Title/Abstract])) OR (university man[Title/Abstract])	97,229
**#9**	(((((Student[Title/Abstract]) OR (School Enrollment[Title/Abstract])) OR (Enrollment, School[Title/Abstract])) OR (Enrollments, School[Title/Abstract])) OR (School Enrollments[Title/Abstract])) OR (((((((((pupil[Title/Abstract]) OR (elementary student[Title/Abstract])) OR (middle school student[Title/Abstract])) OR (junior[Title/Abstract])) OR (senior[Title/Abstract])) OR (high school student[Title/Abstract])) OR (undergraduate[Title/Abstract])) OR (academician[Title/Abstract])) OR (university man[Title/Abstract]))	203,704
**#10**	(relative risk[Title/Abstract]) OR (factors[Title/Abstract])	2,142,135
**#11**	(((("Health Literacy"[Mesh]) OR (Literacy, Health[Title/Abstract])) AND ((China[Title/Abstract]) OR (chinese[Title/Abstract]))) AND ((((((Student[Title/Abstract]) OR (School Enrollment[Title/Abstract])) OR (Enrollment, School[Title/Abstract])) OR (Enrollments, School[Title/Abstract])) OR (School Enrollments[Title/Abstract])) OR (((((((((pupil[Title/Abstract]) OR (elementary student[Title/Abstract])) OR (middle school student[Title/Abstract])) OR (junior[Title/Abstract])) OR (senior[Title/Abstract])) OR (high school student[Title/Abstract])) OR (undergraduate[Title/Abstract])) OR (academician[Title/Abstract])) OR (university man[Title/Abstract])))) AND ((relative risk[Title/Abstract]) OR (factors[Title/Abstract]))	10

Pub.types：article

Database 2: EMBASE

Search	Query	Items found
**#1**	'health literacy'/exp	11,762
**#2**	'literacy, health':ab,ti	207
#3	#1 OR #2	11,832
**#4**	'china':ab,ti OR 'chinese':ab,ti	442,784

**#5**	'student':ab,ti OR 'school enrollment':ab,ti OR 'enrollment, school':ab,ti OR 'enrollments, school':ab,ti OR 'school enrollments':ab,ti	162,473
**#6**	'pupil':ab,ti OR 'elementary student':ab,ti OR 'middle school student':ab,ti OR 'junior；senior':ab,ti OR 'high school student':ab,ti OR 'undergraduate':ab,ti OR 'academician':ab,ti OR 'university man':ab,ti	59,776
**#7**	#5 OR #6	211,097
**#8**	'relative risk':ab,ti OR 'factors':ab,ti	2,774,852
**#9**	#3 AND #4 AND #7 AND #8	4

Pub.types：article

Database 3: Cochrane Library

**Search**	**Query**	Items found
**#1**	'health literacy'/exp	366
**#2**	'literacy, health':ab,ti	2534
#3	#1 OR #2	2542
**#4**	'china':ab,ti OR 'chinese':ab,ti	13,193
**#5**	'student':ab,ti OR 'school enrollment':ab,ti OR 'enrollment, school':ab,ti OR 'enrollments, school':ab,ti OR 'school enrollments':ab,ti	26,610
**#6**	'pupil':ab,ti OR 'elementary student':ab,ti OR 'middle school student':ab,ti OR 'junior；senior':ab,ti OR 'high school student':ab,ti OR 'undergraduate':ab,ti OR 'academician':ab,ti OR 'university man':ab,ti	34,998
**#7**	#5 OR #6	22,662
**#8**	'relative risk':ab,ti OR 'factors':ab,ti	9460
**#9**	#3 AND #4 AND #7 AND #8	28,817
**#10**	'health literacy'/exp	196,268
**#11**	'literacy, health':ab,ti	1

Pub.types：article

Database 4: Web of science

**Search**	**Query**	Items found
**#1**	TS=(Health Literacy or Literacy, Health)	28,783
**#2**	TS=(China or chinese)	2,134,974
#3	TS=(Student or School Enrollment or Enrollment, School or Enrollments, School or School Enrollments)	1,321,454
**#4**	TS=(pupil or elementary student or middle school student or junior；senior or high school student or undergraduate or academician or university man)	1,125,684
**#5**	TS=(relative risk or factors)	11,346,389
**#6**	#5 AND #4 AND #3 AND #2 AND #1	42

Pub.types：article

Database 5: CNKI

(主题=健康素养 或者 题名=健康素养 或者 v_subject=中英文扩展(健康素养) 或者 title=中英文扩展(健康素养)) 并且 ((((((((摘要=学生 或者 abstract_en=中英文扩展(学生)) 或者 (摘要=儿童 或者 abstract_en=中英文扩展(儿童))) 或者 ((摘要=男童 或者 abstract_en=中英文扩展(男童)) 或者 (摘要=女童 或者 abstract_en=中英文扩展(女童)))) 或者 ((摘要=幼儿 或者 abstract_en=中英文扩展(幼儿)) 或者 (摘要=青少年 或者 abstract_en=中英文扩展(青少年)))) 或者 ((摘要=男孩 或者 abstract_en=中英文扩展(男孩)) 或者 (摘要=女孩 或者 abstract_en=中英文扩展(女孩)))) 或者 ((摘要=

小学生 或者 abstract_en=中英文扩展(小学生)) 或者 (摘要=中学生 或者 abstract_en=中英文扩展(中学生)))) 或者 ((摘要=大学生 或者 abstract_en=中英文扩展(大学生)) 或者 (摘要=中小学生 或者 abstract_en=中英文扩展(中小学生)))) 或者 ((摘要=初中生 或者 abstract_en=中英文扩展(初中生)) 或者 (摘要=高中生 或者 abstract_en=中英文扩展(高中生)))) 并且 (((摘要=因素 或者 abstract_en=中英文扩展(因素)) 或者 (摘要=影响因素 或者 abstract_en=中英文扩展(影响因素))) 或者 ((摘要=危险因素 或者 abstract_en=中英文扩展(危险因素)) 或者 (摘要=相关因素 或者 abstract_en=中英文扩展(相关因素)))) (模糊匹配)

期刊论文：462 条结果

Database 6: Wan Fang

主题:(健康素养)*主题:(学生+儿童+男童+女童+幼儿+青少年+男孩+女孩)*主题:(小学生+中学生+大学生+中小学生+初中生+高中生)*全部:(因素+影响因素+危险因素+相关因素)

期刊论文：1047 条

Database 7: CQVIP

((题名或关键词=健康素养 AND (((((((题名或关键词=学生 OR 题名或关键词=儿童) OR 题名或关键词=男童) OR 题名或关键词=女童) OR 题名或关键词=幼儿) OR 题名或关键词=青少年) OR 题名或关键词=男孩) OR 题名或关键词=女孩)) AND (((((题名或关键词=小学生 OR 题名或关键词=中学生) OR 题名或关键词=大学生) OR 题名或关键词=中小学生) OR 题名或关键词=初中生) OR 题名或关键词=高中生)) AND (((题名或关键词=因素 OR 题名或关键词=影响因素) OR 题名或关键词=危险因素) OR 题名或关键词=相关因素))

期刊论文：92 条
